# Mesenchymal stromal cell biotherapy for Parkinson’s disease premotor symptoms

**DOI:** 10.1186/s41016-023-00338-z

**Published:** 2023-10-13

**Authors:** Jinmei Sun, Wei Zhang, Zheng Zachory Wei, Xiaopeng Song, Liu Jian, Feng Jiang, Shuanglin Wang, Haibo Li, Yongbo Zhang, Houzhen Tuo, The CtrLyin Group

**Affiliations:** 1 Clinical Diagnosis and Treatment Center for Parkinson’s Disease, Beijing Friendship Hospital, Beijing, China; 2 Laboratories of Biological Therapeutic Medical Technology, Department of Neurology, Beijing Friendship Hospital Center for Neurological Disorders, Capital Medical University, Beijing, China; 3 National Clinical Research Center for Digestive Diseases, Beijing Friendship Hospital, Neuroscience Institute, Beijing, China; 4 Department of Anesthesiology, Emory University School of Medicine, Atlanta, GA USA; 5 Beijing Tropical Medicine Research Institute, Beijing, China; 6 McLean Imaging Center, McLean Hospital, Harvard Medical School, Belmont, MA USA; 7 Neuroscience Research Institute, Peking University, Beijing, China; 8 Casstar, Zhongguancun No.1 Global Key & Core Technology (AI) Innovation Center, Beijing, China; 9 Department of Critical Care Medicine, Airport Hospital of Tianjin Medical University General Hospital, Tianjin, China; 10 Department of Cardiovascular Thoracic Surgery, Tianjin Medical University General Hospital, Tianjin, China; 11 Department of Biochemistry and Cell Biology, Geisel School of Medicine, Dartmouth College, Hanover, NH USA

**Keywords:** Cell therapy, Cognitive impairment, Depression, Neuronal survival and differentiation, Parkinson’ s disease

## Abstract

Parkinson’s disease (PD) is a neurodegenerative disorder with motor deficits due to nigrostriatal dopamine depletion and with the non-motor/premotor symptoms (NMS) such as anxiety, cognitive dysfunction, depression, hyposmia, and sleep disorders. NMS is presented in at least one-fifth of the patients with PD. With the histological information being investigated, stem cells are shown to provide neurotrophic supports and cellular replacement in the damaging brain areas under PD conditions. Pathological change of progressive PD includes degeneration and loss of dopaminergic neurons in the substantia nigra of the midbrain. The current stem cell beneficial effect addresses dopamine boost for the striatal neurons and gliovascular mechanisms as competing for validated PD drug targets. In addition, there are clinical interventions for improving the patient’s NMS and targeting their autonomic dysfunction, dementia, mood disorders, or sleep problems. In our and many others’ research using brain injury models, multipotent mesenchymal stromal cells demonstrate an additional and unique ability to alleviate depressive-like behaviors, independent of an accelerated motor recovery. Intranasal delivery of the stem cells is discussed for it is extensively tested in rodent animal models of neurological and psychiatric disorders. In this review, we attempt to discuss the repairing potentials of transplanted cells into parkinsonism pathological regions of motor deficits and focus on preventive and treatment effects. From new approaches in the PD biological therapy, it is believed that it can as well benefit patients against PD-NMS.

## Background

In brain degenerative conditions on the middle-aged or the elderly ages, Parkinson’s disease (PD) occurs with progressively worse motor defects such as bradykinesia, myotonia, resting tremor, and rigidity and develops a wide range of non-motor/premotor symptoms (NMS).

Parkinson’s associated symptoms mainly relate to the diseased and pathological changes in the brain [1]. Their PD-NMS convergently develop autonomic dysfunction, cognitive and mental impairments, sensory disturbances, and/or sleep disorders.

NMS can appear earlier than PD motor dysfunction and fluctuate with the motor symptoms later. For example, among approximately half of Parkinson’s patients, there are anxiety (a total of 40 percent) and/or depression, which are presented years before typical motor PD symptoms occur, while PD-associated mood and sleep disorders usually develop during the course of PD [2]. Use of recent tools of the non-motor symptom questionnaire (NMSQuest) or the non-motor symptom scale (NMSS) has been helpful for PD-NMS evaluation and the quality-of-life improvement. In addition to medication, the surgical treatment can be given based on symptoms of the disease.

Stem cell trials with surgical procedures have been proposed as a viable treatment option in Parkinson’s patients [3]. Mild cognitive decline is another common NMS presented in one-fourth PD, with domains affected in the attention and working memory, bradyphrenia, the executive function, and/or the visuospatial function [4]. Our group recently focuses on the stem cell potentials for the cognitive recovery and the further effectiveness of other mental illnesses [5]. UC-MSC or embryonic cells may be considered as one of the efficient type in the clinical use considering their relatively naive ages [6]. We discuss the potential use of type of their derived stem/progenitor cells, biological characteristics, and the efficacy for the application in PD.

### PD-associated non-motor symptoms (PD-NMS), symptomatic relief, and medications

Therapeutic effects on motor improvement have been related to the protective maintaining of a relative balance for acetylcholine/dopamine in the brain regions. In addition to problem-solving of motor fluctuations using the oral levodopa medication or the surgical deep brain stimulation alone, increased trends of incidence/prevalence rates of PD-NMS are demonstrated [7]. We review PD-NMS treatments for the understanding of current status with an attempt to improve symptomatic relief via new biotherapies including the stem cell and probiotics implant approaches (Table 1).

**Table 1:** Neuropsychiatric and early PD/pre-PD treatments

Symptom	Clinical intervention		Potential biotherapy		Other recommendation
		**Risk**		**Delivery path**	
Anxiety	Benzodiazepines (diazepam, lorazepam)	Lead to chronic stress that can worsen NMS	-	-	Further gut-brain axis modulation (gut microbiota with *Lactobacillus plantarum* PS128)
Cognitive impairment	Donepezil, galantamine, rivastigmine	Development of dementia	BMSC	Intravenous infusion	Increased adverse events (accidental falls, cognitive deterioration) with antipsychotics
Depression	Cognitive behavioral therapy, pramipexole, repetitive transcranial magnetic stimulation, venlafaxine	Prolongation of the Q-T interval when citalopram exceeds 20 mg in patients over 60 years old	NSC	Intravenous infusion	Sertraline, paroxetine, fluoxetine, citalopram with mild adverse reactions, contraindicated with monoamine oxidase B inhibitors MAO-BI. ECG monitoring is recommended Hippocampal/NSC neurogenic compound NSI-189
Psychosis	Clozapine, pimavanserin	-	UC-MSC	Intravenous infusion	Regular blood routine monitoring is required
Impulse control disorder	Cognitive behavioral therapy	-	-	-	Dopamine agonist withdrawal syndrome should be monitored
Indifference	Piribedil	Comorbid depression, caregiver burden	-	-	Monitor patients for apathy after subthalamic nucleus deep brain stimulation surgery
Orthostatic hypotension	Droxidopa, fludrocortisone, midodrine	-	-	-	Moderate the use of diuretics, antidepressants, etc
Chronic constipation	Polyethylene glycol, probiotics/prebiotic fiber	-	Fecal microbiota transplantation	Upper gastrointestinal tract via nasoenteric tubes	Gut microbiota with probiotic supplements [8]
Anorexia, nausea, vomiting	Domperidone	-	-	-	Electrocardiogram needs to be monitored
Salivation	Ultrasound-guided botulinum toxin type A/B the injection to parotid gland/ submandibular gland	-	-	-	Chewing
Urinary dysfunction	Solifenacin	-	Myoblasts	Intrasphincteric injection	Anticholinergic (dry mouth, constipation) and non-cholinergic (indigestion, dizziness, headache) adverse reactions
Erectile dysfunction	Sildenafil	-	ASC	Intravenous infusion	Monitor orthostatic hypotension
Insomnia	Amantadine, eszopiclone, melatonin	-	-	-	Moderate the use of anti-PD drugs; continuous positive airway pressure for obstructive sleep apnea; further gut-brain axis modulation (gut microbiota with *Lactobacillus fermentum* PS150)
RBD	Clonazepam, melatonin	-	-	-	Moderate the use of SSRIs, SNRIs, TCAs, MAO-BI, or benzodiazepines
Pain	Opioids	-	-	-	Monitor constipation, gut microbiota with probiotic supplements, MSC-derived exosomes injection
Fatigue	Rasagiline	-	-	-	-

Scales currently used to assess NMS in Parkinson’s patients include NMSQuest, NMSS, International Parkinson and Movement Disorder Society (MDS) unified PD rating scale (MDS-UPDRS), and other questionnaires/scales of symptom-specific assessment. NMSS has been widely used in clinical practice/trials since 2005, but with limitations, such as in anxiety, apathy, and depression, for the same domain, in fatigue and sleep disturbance for the same domain, missing drug-induced problems of non-motor fluctuations, and impulse control disorders with limited cognitive domains. The MDS-NMS scale since 2015 includes 52 items, involving 13 areas, and assessing the severity and frequency of NMS in Parkinson’ s patients with a score of 0 to 4. The subscales of this scale include eight items, which evaluate the degree of NMS changes related to the anti-PD drug treatment on a scale of 0 to 4. MDS-NMS scale is shown with the statistical analysis data with a valid measure of frequency of assessing impulse control disorders and related disorders, assessing cognitive and other neuropsychiatric aspects of PD. The non-motor fluctuations subscale assesses NMS in PD and the effect of fluctuating treatments

For PD neuropsychiatric symptoms of anxiety and depression, selective serotonin reuptake inhibitors (SSRIs)/antidepressants (approved to treat anxiety, depression, and other mood disorders) have included citalopram (Celexa), escitalopram (Lexapro), fluoxetine (Prozac), fluvoxamine (Luvox, Luvox CR), paroxetine (Paxil, Paxil CR), sertraline (Zoloft), and vilazodone (Viibryd). In PD combating depression (due to dysregulation in the brain areas of producing dopamine, norepinephrine, or serotonin), SSRIs may aggravate tremor in 5–20% of Parkinson’s patients. Amitriptyline that is considered clinically useful but with adverse reactions of anticholinergic side effects drives particular attention in cardiac arrhythmia or cognitive decline according to the guideline. In Parkinson’s patients with hypertension and/or diabetes, it is necessary as well to prevent and treat cerebrovascular disease-related cognitive impairment (Table 2).

**Table 2:** Representative stem cell trials and designed research for PD and some other neurodegenerative disease in patients

Potential bio-therapy intervention		Study design information	
**Cell source**	**Delivery path**	**Treatment number/test(s)**	**Report year(s)**
Embryonic dopamine cell	Implant surgery	Total 34 cases/(NCT00038116)	2001–2009
ESC-derived A9 dopamine progenitor cell	Administration of the putamen into the brain (~ 10^6^)	Twelve patients/(NCT05887466)	2001–2023
Autologous adipose tissue-derived stromal vascular fraction	Subdermal plane injection (~ 10^7^)	Ten patients/inflammatory biomarkers at 12-month follow-up (NCT05699161)	2009–2020
ESC-derived neural precursor cell	Striatal implantation	Total 50 cases/(NCT03119636)	2018
RC17 ventral midbrain dopaminergic progenitor cell	Surgical implantation (10^6^–10^7^/six tracks)	Eight patients/HLA class I detection (NCT05635409)	1989–2020
Autologous peripheral nerve graft	Unilateral implantation into the substantia nigra	Eight patients/deep brain stimulation therapy reduction (NCT01833364)	2016–2018
Neural stem cell	Nasal cavity delivery (10^6^/week)	Twelve patients/(NCT03128450)	2013–2014
Autologous peripheral nerve graft	Implantation into the substantia nigra, basal forebrain, putamen with/o STN-DBS	~ 70 patients/DaTscan SPECT imaging assessment at up to 24-month follow-up (NCT02369003) [9]	2005–2022
Parthenogenetic neural stem cell	Intracerebral injection into striatum and substantia nigra	Twelve patients/(NCT02452723)	2016–2018
Autologous adipose-derived MSC	Intravenous injection	~ 30 patients/(NCT04995081)	2005–2020
Bone marrow-derived MSC	Intravenous injection	Twenty patients/(NCT01446614)	2007–2010
Allogeneic bone marrow-derived MSC	Infusions (10^7^/kg)	Thirty cases/(NCT04506073)	1996–2018
Autologous MSC	Intravenous intervention	Thirty patients/(NCT04146519)	2020
Autologous bone marrow-derived MSC	Intravenous route and intranasal route into inferior nasal conchas and meatuses	~ 500 patients/(NCT02795052)	2011–2016
Topical bone marrow-derived MSC fraction	Intravenous and intranasal routes (14 cc and 1 cc)	~ 100 patients/(NCT03724136)	2009–2018
Autologous adipose-derived tissue stromal vascular fraction	Intravenous parenteral route (500 cc)	~ 300 patients/(NCT03297177)	1988–2015

One typical inclusion criterion may be from idiopathic PD of at least 7 years duration and who is responsive to levodopa. Moderate-to-severe cognitive impairment and depression are normally excluded. The screening visit during an enrollment can be based on participants’ MDS-UPDRS parts II and III scores data. Immunologic responses and serious adverse reactions are some of important safety and tolerability measurement. The summaries include any approved, withdrawn, or registered clinical study representatives with reference number provided

There are anti-PD drug-associated psychiatric symptoms (inducing PD psychosis) in patients with the anticholinergic drugs, amantadine, MAO-BI (such as (lazabemide, rasagiline, or selegiline), dopamine agonist, and levodopa. For an adjustment of unsatisfactory anti-PD drugs, low-dose quetiapine treatment is proposed with the mechanisms of histamine H1 and alpha-1 adrenergic receptors that may have negative metabolic effects and significant adverse effects. Dementia/cognitive impairment can be presented with the influence of anti-PD psychotic drugs. For protecting the cognitive function, use of trihexyphenidyl and other anticholinergic drugs should be avoided. Many other common situations also include an impulse control disorder usually identified in Parkinson’s patients with dopamine agonist, which can become significantly worse with the higher dose of levodopa [10]. Meanwhile, rivastigmine is added for an indifference that occurs with apathetic symptoms during optimizing anti-PD drugs. Practically, to reduce autonomic dysfunction using nondrug clinical interventions, there are suggestions on increasing water and salt intake, raising the head position during sleep, avoiding rapid body position changes, wearing elastic pants, etc., against the orthostatic hypotension or the chronic constipation (intake of sufficient fluid and fiber, appropriate exercise while stopping or reducing anticholinergic drugs for chronic constipation). Overcoming drug-related anorexia, nausea, and vomiting, it is commonly operated when levodopa is given with some snacks before the main meal, or the medicine taken 0.5 to 1 h after the main meal and 2 h after the other meal for the symptoms still exists after 2 weeks. Relatively, there are PD-NMS urinary dysfunction (exclude other related diseases such as in male prostate disease, female pelvic floor disease, urinary tract infection) and PD-associated erectile dysfunction other than drugs or related diseases (such as depression, prostate disease, and diabetes). For related treatment of urinary incontinence, recent trials of autologous MSC have been demonstrated promising [11]. Our group is also testing discovery mechanisms of the biotherapy for replacing fecal microbiota transplantation with gut probiotics into Parkinson’s patients [12].

In patients with concurrent insomnia/sleep-wake disorder, the use of levodopa controlled-release, long-acting dopamine agonist (rotigotine transdermal patch, etc.) or sleep hygiene consultation may be related to an excessive daytime sleepiness/sleep attack. With clarification for the cause of excessive daytime sleepiness, the patient develops drowsiness after each dose of dopamine agonist [13]. Additionally, depression, insomnia, or obstructive sleep apnea disorders may still be induced in the conditions, while modafinil is normally used.

Parkinson’s patients develop rapid-eye-movement (REM) sleep behavior disorder (RBD) by a specific loss of normal muscle atonia in REM sleep. There is currently a lack of RCT studies on PD-NMS RBD treatment. In the many clinically supportive researches, presentation of RBD indicates a neurodegeneration progression, but it needs to be excluded for RBD induced by some medications [14]. Due to muscular dystonia in the early and chronic PD, there are pain (exclude other causes of pain, such as osteoarthritis, etc.) and pain-related fluctuation of symptoms, when anti-PD drugs can be adjusted to prolong the “on period” and improve the “off period” pains.

Fatigue identification and clarification with PD have been performed. Our group recently test an acute exercise-induced fatigue scale (AEIFS) as an evaluation for the fatigue chronically developed [15]. The patients who are able to take acute exercises can be quantified for assessing fatiguing conditions [16]. It is useful for PD-NMS fatigues which can seem invisible to be explained [17], whereas the peripheral and central fatigues become comorbidity conditions with the muscle stiffness, changes in the ability of sleep/walk, the slow movement, and many others. When treatment, it needs/helps in considering the possible factors in the cause of fatigue.

### Stem cell transplantation, motor improvement, and therapeutic strategy

It may have been evidenced in promoting the renewal of cardiomyocytes, well-controlled blood sugar, repairing wounds or restorative neurological/behavioral functions, etc. for BMSC (commonly blood sources) and UC-MSC (to be obtained from collected umbilical cord tissues) [18]. Additionally, there are several technical advantages (available sources, simplified separation/purification protocols, and noninvasive operations as invasive bone marrow puncture for BMSC becomes not necessary in recent method). Among them, the hUC-MSC possess high native self-renewal and strong proliferative ability with low immunogenicity. Supportively, our understanding of the efficient transplantation requires identification of little immune rejection (via suppressive local microenvironment with indoleamine 2,3,-dioxygenase, interleukin-10, and prostaglandins pathways) or weak reaction (absent from MHC-II or costimulatory molecular mechanisms) [19]. Furthermore, cell research from field experts have changed/redesigned allotransplantation with the relative capacity to introduce foreign genes [20].

An ideal strategy in brain diseases would be taking advantage at full capacity of an efficient use for cell therapy, of an application to the source selection considerations on neural cells (including glial cells)/vascular cells, and of a biological and physiological tone in the posttransplantation host microenvironment [21]. For preventing neurotoxic spreading, successive anti-α-synuclein immunotherapy seems to exert beneficial efforts with prasinezumab for slowing motor decline in participants subgroups of recognizable fast progression. Other traditional treatment methods can neither reverse neurodegeneration nor restore number of dopaminergic neurons [22]. Theoretically, neuronal cell regeneration and repair of damaged/dead tissues remain ideal methods for the treatment of PD [23]. Likewise, grafted embryonic-derived premature cells have been demonstrated an ability in survival, growth, and function until at least 36 months later targeting an ongoing diseased process of the degeneration. The putamen fluorodopa uptake in the transplantation regions is further increased. A number of transplant recipients can develop their robust innervation of dopaminergic grafts with transplanted cells into the striatum [24]. Together with positron emission tomography or by autopsy, there are signs of a partial improvement in parkinsonian motor regions. How neuronal repair renders the element of neuromodulation and benefits patients with centrally related PD-NMS has not yet been discovered [25].

In a general example for a mesenchymal stromal cell, given stronger plasticity, lower immunogenicity, and higher cell variability (due to faster in vitro expansion and easier induction with potential carrier of foreign genes), it becomes one of important sources considering its targeted tissue microenvironment. Therapeutically, lumbar puncture or subarachnoid injection of the stem cells can be achieved in 3rd /4th lumbar intervertebral space [26]. The related clinical symptoms have been evaluated for bradykinesia, postural instability, rigidity, or tremor to be significantly improved (wumedicalcenter.com), which may be consistent with an average rate of 38% for clinical improvement among all patients followed up at 36-month post-transplant in other reports. Experimental transplantation of hUC-MSC-derived tyrosine hydroxylase cells into the striatum tracing in a rat model of PD shows enhancing long-term cell survival with no longer than 4 months, for the cells can migrate within a range of 1.4 mm from the transplantation site into the deeper tissue [27]. Consistently, the amphetamine-induced turning behavior of the cell-treated rats is significantly recovered, which is quantitatively used in long-term assessment of motor improvement [28].

Another example from earlier research includes eight Parkinson’s patients receiving a therapy of MSC injection through carotid artery, with similar design tested in animal models [29]. The results show that their daily activities and motor symptoms, rigidity, tremor, and/or the unified PD rating scale (UPDRS) scores are significantly improved (cjter.com). With injection of MSC into the subarachnoid space alternatively reported in 38 Parkinson’s patients, whose UPDRS at 1 month later are significantly improved as well, there are few adverse immune reactions during the follow-up period (some reporting an 80% improvement in motor functions/skills until 36 months later). In China, the recent application to the stage of stem cell clinical trials for PD starts with focus on cognitive/psycho-emotional improvement and PD-NMS [30].

Enhancement strategies of cell-based mechanisms have been investigated in past 10 years for the design that particularly uses transgene vectors committed to novel stem cell transplantation therapies [31]. To overcome low survival/retention rate or insignificant long-term efficacy of the treatment supposedly achieved field breakthrough for neurological disorders, studies using that stem cell as a carrier of gene therapy combine its curative effects, e.g., which are modified in vitro with transcriptional regulation factor (such as by Nurr1 of nuclear receptor superfamily, human gene name: *NR4A2*). Nurr1 is believed to become the differentiation initiator of neural progenitors into dopaminergic neurons eventually [32]. With induction protocols, transplantation of dopaminergic neurons divergent from the Nurrl gene-modified stem cells into a PD rat model may be more efficient to promote the expression of dopamine and tyrosine hydroxylase content, as evidenced in the rat brain. A line of tyrosine hydroxylase-overexpressed stem cells transplanted into the lateral ventricle of rats seem to show higher cell viability and migrating capacity as well as better survival compared to ones in control conditions. Many previous and recent studies have supported an integration critical to both the cellular phenotype and behavioral effects [33, 34]. Immunohistochemistry has supported an enhanced positive expression of tyrosine hydroxylase (that is supposed to produce additional dopamine) in injections originally modified with tyrosine hydroxylase gene. The dopamine content in the striatum of PD rats can be significantly increased by the transplantation, indicating that stem cells combined with gene therapy can provide more effective therapeutic potentials than transplantation alone [35]. Other than the introduction of foreign genes and genetic modification along with the injections (such as different methods with increased expression to induce dopamine as an intervention for PD), we and other groups have tested more robust preconditioning strategy on cells for biotherapeutic applications. Specifically, pre-treatment strategy prior to transplantation with any donor cells using pleiotrophin (priming of the growth factor) may improve the PD-related motor function (rodent rotation behaviors). Aforementioned, it has been revealed for the promoted survival of tyrosine hydroxylase-positive grafts as a direct mechanism of potentially enhanced therapeutic effects [35].

### PD mechanisms, biotherapeutic microenvironment, and targets

For PD (and other) neurodegenerative disease treatments, research to date can identify neurotrophic mechanisms behind promotion of neurological recovery by mesenchymal stromal cell transplantation [36]. MSC that reach the regenerative microenvironment create a favorable regeneration niche of functional neurons by secreting a variety of growth factors, produce critical extracellular matrix proteins, and are laterally thought to give effective neuromodulation. Their paracrine potentials for the protective factors into the microenvironment have included BDNF, IL-6, neurotransmitters, NT3, SCF, and VEGF [37, 38]. With respect to PD treatment strategy of rescuing dopaminergic neurons, the type of stem cells normally promote angiogenesis and exert anti-inflammatory and anti-apoptosis effects and improve the survival rate at the graft periphery [39]. In addition, an extracellular matrix modulation may occur at the transplantation site, which demonstrates the ability of supporting endogenous neural cell attachment and growth, differentiation, and neurogenesis, involved in the tissue repair and functional recovery [40]. Researchers assume that factors from transplanted cells are responsible for protection/recovery of a dopaminergic neuronal function and for their primary therapeutic effect on parkinsonism conditions.

**Fig. 1:**
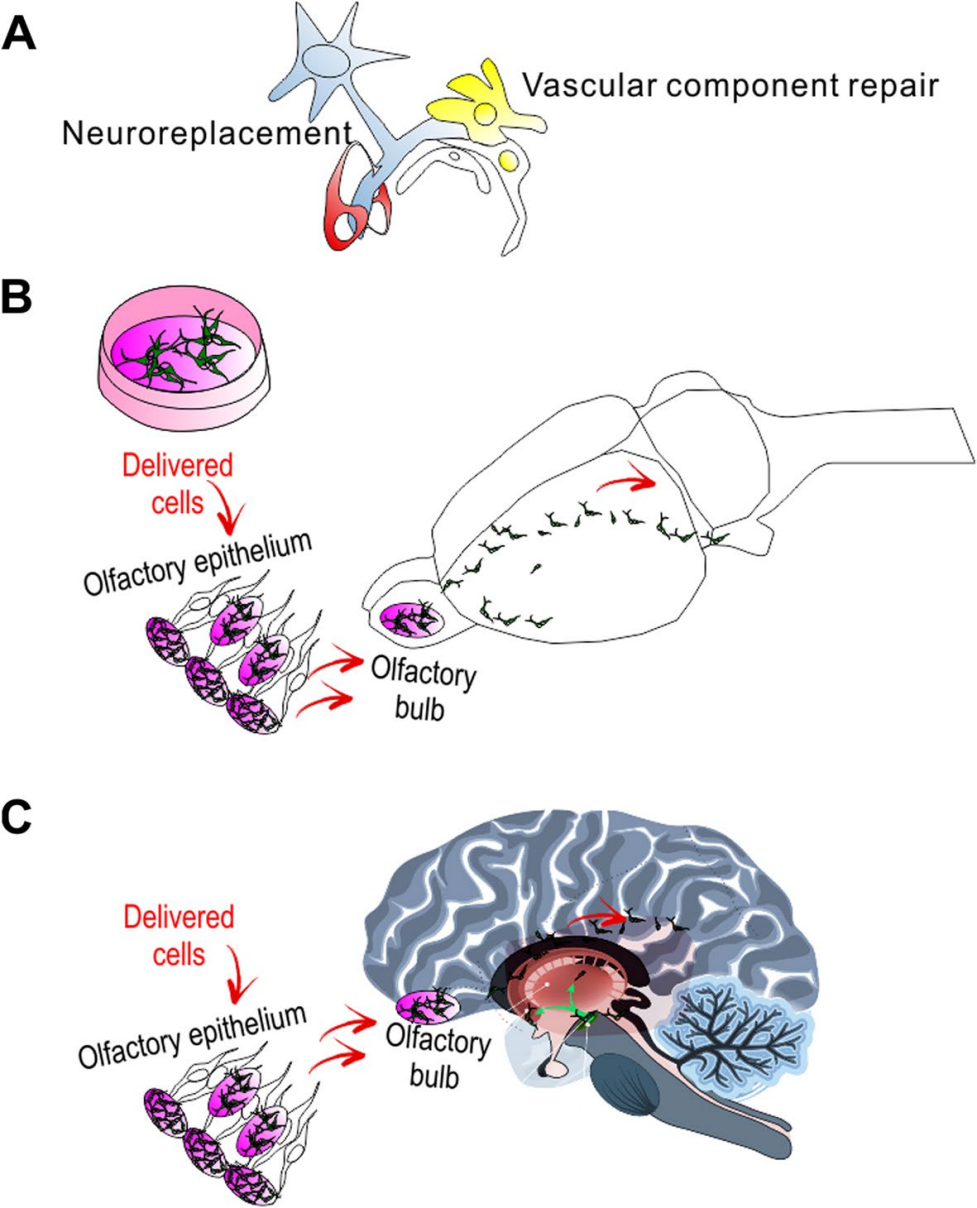
Schematic diagram of cell transplantation treatment. With the potentials of exerting multiple functions, the transplanted cells enter the brain, migrate to the injured site, and transform into neuron-like cells, directly replacing damaged/necrotic neurons surrounding non-injured nerves. Knowledge on stem cell mechanisms based on neuroprotective effects is summarized but still limited. Additionally, MSC have a strong paracrine effect, secreting or promoting BDNF, GDNF, VEGF, and other active substances [42]. The retained cells are expected to promote the survival, proliferation, and differentiation of new progenitors, promote the formation of new blood vessels, migrate to the injury site, and help to engage the neural circuit by promoting the formation of synapses

In an improved microenvironment, cell replacement effect with the transplanted stem cells may be still dependent on a differentiation into dopaminergic neuron-like cells or activated endogenous neural stem cells to replace corresponding types of neural cells in the damaging dopaminergic tissues [41]. It should be noted in various studies for a cell replacement as the primary goal and main mechanism of stem cells. Without enhancement strategies, the retention rate is usually less than 5%, or their number will rapidly decrease following original injection. Whether controlled precise differentiation alone can restore neurological functions through its integration still awaits further research. The handbook for stem cell biology from State Key Laboratory of Stem Cell and Reproductive Biology as well as from the other laboratories is published with focus on direct differentiation into functional units, distributed in Chinese Edition. Furthermore, MSC highlighted in vascular mechanisms of stem cells, such as the neurovasculature and gliovascular units (Fig. 1), need to be revisited.

Autopsy has indicated identifiable activation of microglia from PD brains in the postmortem study where both local and systemic inflammation (e.g., spreading through the periphery—the vagus nerve-brain) can induce the proliferation effect of microglia. Transplanted particular stem cell type should inhibit kinds of proliferation triggers with reduced expressions of microenvironmental pro-inflammatory factors. Elevated levels of the pro-inflammatory cytokines detected in the brains of Parkinson’s patients have included IFNγ, IL-1β, and TNFα [43]. Another example is that there is high fecal calprotectin marker detected from Parkinson’s conditions due to elevated intestinal inflammation. Facing the established systemic inflammation and responses, transplantation studies have reported that the immunosuppressive cyclosporin A for the PD animal models are needed. It illustrates the importance of the immunomodulatory and anti-inflammatory effects needed by stem cells in the PD treatment. Since, recent research for stem cells provide promising reparative strategies in the forefront of PD research and some new cell trials (the cells keeping the main advantages, e.g., noninvasive homing, without ethical disputes, and with low immunogenicity). Standardized stem cell bank (such as the mesenchymal stem cell bank), with the understanding of the biological characteristics and developing clinical aspects, has been established given the future possibility of the restorative method for the treatment of PD [44]. How to obtain a most efficient treatment following transplantation planning for neuroprotection by stem cell, as well as the transplantation time window, controllable cell number, practical pathway, long-term safety, and risk, still needs further investigation on individual precision and evaluations on the microenvironmental factors [45].

The need for cell replacement with dopaminergic neurons to the Parkinson’s patients gradually increases over time during disease progression (xellsmart.com). Under the advanced use of small molecule series combined with certain circumstances, adult cell (including stem cell) can be expected reprogrammed towards specified stage of neurons [46]. In the PD animal models, intracranial stem cell transplantation stimulates an increase in the total number of dopaminergic neurons with consistent functional recovery. Whether the transplanted cells that are targeted transforming into dopaminergic grafts can become parts of physiological integration and neurogenesis still need to be verified in vivo (e.g., in nonhuman primates). Transplanted green fluorescent protein (GFP)-labeled trabecular meshwork MSC into an animal model of PD with potential GFP/tyrosine hydroxylase-double positive cells retain leaky GFP expression issues that need to be overcome [47]. However, transplantation of differentiated hUC-MSC can show the dopaminergic expression of tyrosine hydroxylase with well cell retention which is expected beneficial from cellular activity, instead of a focus on the dopaminergic neuronal integration. On the other hand, bioadhesives may greatly improve an adhesion effect of cells and additional microcarriers of neurotrophin-3/polylactic-co-glycolic acid (PLGA) helpful to the directed dopaminergic induction [48]. In order to improve the clinical efficacy of the differentiating cells, researchers from our and many other groups also test one of the simple and robust ways in regulation of physiological/pathobiological microenvironment, in which hypoxic/inflammatory mimic conditions can also promote the differentiation/adaptation of stem/immature cells [49]. On the other hand, olfactory ensheathing cells combined with OM-MSC may become sources of regenerative dopaminergic nerves. Additionally, preconditioning with olfactory ensheathing cell-growth medium can significantly upregulate hypoxia adaptation factor HIF-1α in the tissue and cells.

Researchers have long reported in studies using immunosuppressants to treat animal models of PD. Immunomodulatory effects on the increased levels of pro-inflammatory cytokines (e.g., TNF-α, IL-1β, and IFN-γ) have been observed in the Parkinson’s patient brains with a large number of proliferating activated immune cells [50]. For example, the PD-associated astrocytes within the pathologic brain are primarily involved in functional major histocompatibility complex class (MHC)-II receptors for the role of CD4 T-cell activation upon antigen presentation. In pathological PD conditions, there are excessive astrocytic α-synuclein accumulation and can be spread with adjacent cells along with the pathological propagation, where mature T cells are required with an activation of MHCI/II, derived into specific subtypes of CD8 + cytotoxic T cells and CD4 + T-helper cells. Recent studies have also shown that α-synuclein in patients with PD can be attacked by their autoimmune system as an antigen. That indicates one pathogenic mechanism of PD closely related to the immune system, which our group has recently tested in a registered clinical trial (NCT04871464). From the point of view of cell biology, it is shown for MSC by the advantages of exhibiting a main immune-regulatory response and immune suppression effects, responding under Toll-like receptors, downregulating innate and adaptive immunity, and exerting immunoregulatory functions (immune-enhancing or immunosuppressive properties) through direct cell-to-cell contact or secretion of soluble factors (IL-17, TGF-β1, etc.), facilitating Th2/Tregs inductions. In addition, MSC may prevent an activation of microglia in addition to dendritic cells, increase the level of anti-inflammatory factors (IL-10, PGE2, etc.), promote the autophagic clearance of pro-inflammatory α-synuclein, and reduce the autophagy and oxidative stress of cells including that with SIRT2/SIRT4 activation.

In PD and other neurodegenerative conditions as one combinatorial target, their glutamate-induced excitotoxicity [51], a reactive oxygen species overproduction [52], and DNA repair defects [53] are critical factors leading to the neuronal apoptosis of chronic neurodegenerative processes. Normally, stem cells can activate those endogenous defense/repair mechanisms by paracrine release, responsible for reducing the loss of dopaminergic neurons. In 6-hydroxydopamine-induced PD models, it is demonstrated that exosomes from stem cells inhibit damaging responses caused by mitochondrial dysfunction through AKT, ERK, and p38 pathways to promote neuroprotection [54]. A variety of paracrine factors (GDNF, NGF, NT-3, VEGF, etc.) are confirmed. Furthermore, applied with enhanced biotherapeutic approaches in overexpressing GDNF into the stem cell, there are significant increases for tyrosine hydroxylase-positive staining of the striatal regions following transplantation [55]. We believe that the trophic support via growth factors and protective cytokines is achievable for the treatment of the conditions. The reports have included prostaglandin EP2 receptor downstream, MAPK and PI3K/AKT signaling effects. More importantly, additional cells provided by transplantation usually demonstrate acceptable potentials of retention and conditioning to achieve therapeutic goals in PD. For example, probability of observing arterial repair (vascular component repair) and promoted angiogenesis in the PD conditions can be further increased for human MSC [56]. In the studies, inhibiting the expression of chemokine CXCL-9 is able to enhance an angiogenic capability of the cells, via ERK1/2 MAPK and PI3K/AKT pathways, for both angiogenesis and subsequent neurogenesis.

In patients with PD, cell metabolism can be improved by mitochondria (the energy factories for the cells) as a means of improving processes for cell oxidative phosphorylation, energy supply, cell differentiation, oxygen free radical formation, and apoptosis control. Toxic substances or mutations in mitochondrial DNA may disrupt the oxidative respiratory chain within the mitochondria. Theoretically, those can lead to the damage of the oxidative respiratory complex function in the target region/transplantation site of diseased conditions, which aggravate oxidative stress and cause damage to dopaminergic neurons. Fortunately, transplanting stem cells has demonstrated the ability of alleviating those mitochondrial dysfunction [57]. Via transferring of normal mitochondria, these stem cells (e.g., HSPC, MSC cell types) reduce the potential for challenges in damaged endothelial cells and related progenitors, improving microvascular structure and microenvironment. Somehow, it may be then hoped that transplanted stem cells provide normal mitochondria for neurons, to maintain normal metabolism and inhibit oxidative stress/neuronal apoptosis (in combination with intravenous, intrathecal, intranasal, or another path). The issues to be confirmed are as follows: What the best way and how efficient is it for the delivery? How to prevent the safety risk, immunogenicity and ethical issues of allogeneic treatment if the cells are applied in the clinic? It is agreed that the most suitable seed cells for transplantation should be selected from the bank based on higher levels of safety characteristics, strong proliferation, and differentiation ability [58]. We and other groups have also attempted to focus on vascular functional and microenvironmental evidence by the transplantation therapy and mechanisms [59–61].

### Psychological pre-PD/PD-NMS, hippocampal involvement, and interventions

Depression, mainly characterized by significant and lasting depressive symptoms, is a common mental illness that endangers the physical and human mental health. The WHO reports that about 5% of the world’s population (about 340 million in total) are suffering from depression. Unfortunately, the first-line treatment of depression with antidepressants of SSRIs, SNRIs, NaSSA, and SARIs shows limited effects for relieving symptoms in depressed patients. Pre-PD depression (a history of depression prior to an onset of PD motor symptoms) demonstrates an association with poorer cognitive abilities. As of 2020, depression may become the world’s second-most common disease after heart disease. PD-NMS and the depression history are detectable and correlated to the Beck Depression Inventory (BDI) and the Hamilton Anxiety Rating Scale (HAM-A), by which an incidence of depression in women is twice more than that of men. For depressive disorder, it is the main health trend of its global burden of disease study for women aged 15 to 44. Depressive symptoms not only cause a decline in the quality of life of affected individuals and a deterioration in their social functions but also lead to increasing expenditures on health service resources [62]. Since 2006, the annual economic cost of depression burden in China has already exceeded 60 billion Chinese yuan. Among the nonfatal global burden of disease study data, it ranks first for the depression or does in the top high morbidity, high recurrence rate, and high disability rate [63]. PD-NMS depression is associated with caregiver burden/disability rate increase and to reduction in the quality of life. With the “broad spectrum” antidepressant of current medication relatively unsatisfactory, easy to relapse with chemical drug, developing drug resistance, and certain side effects of long-term use, depression becomes a difficult condition to treat. There has been PD-associated treatment for depression via selective serotonergic reuptake inhibitor amitriptyline, citalopram, desipramine, fluoxetine, nortriptyline, paroxetine, sertraline, or venlafaxine and the cognitive-behavioral therapy (CBT) in clinic. It should be noted that currently considered first-line treatment still needs revealing evidence for Parkinson’s patients with depression. Stem cell-based therapy is yet to become new ways to improve depression in the PD conditions [64]. On the other hands, the occurrence of depression is closely related to hippocampal neuronal regeneration dysfunction involving stem cell changes underlying functional alternations. The etiology and pathogenesis of pre-PD/PD-NMS depression have not yet been fully elucidated. As a highly heterogeneous disease, an impairment of neurotransmitter modulation in the brain, neuronal apoptosis, an immune inflammatory response, neuronal degeneration, impaired inferior colliculus, brain-pituitary-adrenal cortex (hypothalamic pituitary adrenal, HPA) axis dysfunction, and many other environmental/genetic mechanisms are related to the pathogenesis of pre-PD/PD-NMS depression.

Indeed, many kinds of antidepressants promote proliferation of the adult hippocampal neuroblast and further upregulate neurotrophic factor expression of BDNF, VEGF, and some others. It is usually maintaining for the curative effect of antidepressants in several weeks since the start of treatment and supporting hippocampal neuronal regeneration as the basis of their encouraging activities [65]. Due to proliferative functions, it leads to hippocampal dentate gyrus activity resulting from 20 to 50% involvement of a total cell population, dependent with antidepressants on different pharmacological mechanisms (similar with electrical convulsion stimulation). Among those, 75% are demonstrated the ability in further neuronal development. Evident in rodent models, development of treatments on neuroprotection, and recovery of neurons are expected primarily responsible for the relief of depressive symptoms. This effect may be independent on the adjustment of the HPA axis (useful prognostic hallmark of depression) in PD conditions. PD is known to be associated with hippocampal atrophy, while the results of research on hippocampal volume show that the volume in depressed patients is smaller. Hippocampal atrophy has been associated with number of depressive episodes and to the length of time without treatment against symptomatic depression [66]. We and some others believe that the selective and sustained loss of hippocampal volume is caused not only by the apoptosis of hippocampal neurons but also by decreased neuronal regeneration [67]. Both neurons and stem cells for the hippocampal neuronal regeneration are impaired underlying the chronic changes.

Since the neuronal damage in the hippocampus is pathologically significant and extremely sensitive to stress, the dentate gyrus should become a unique therapeutic target for the depression as well as in PD with impaired cognitive performance. Taking into consideration of cognition pathways across dentate gyrus and other parts of the hippocampus, the regeneration within these functional neuronal circuits can be constructive. By location, the granular cells in the dorsal dentate gyrus can significantly inhibit contextual learning activities, while the granular cells in the ventral dentate gyrus can inhibit the anxiety and other stress reactions [68]. Typically, dorsal hippocampus is involved in cognition-related behaviors while ventral hippocampus for emotion-related behaviors through the HPA axis. Dorsal dentate gyrus, ventral dentate gyrus, and intermediate dentate gyrus are divergently regulated. When the neuroendocrine system is frequently activated upon various stresses, they have, microglial cell-mediated neuroinflammation and immune activation in the brain, been closely related to the development of depressive symptoms. Systematically, activation of inflammatory cells leads to releasing of a large variety of inflammatory cytokines, as observed for high cytokine/immune factors (IL-1β, IL-6, IFN-α, and TNF-α, etc.) in the peripheral blood of patients with depression [69].

Our aforementioned hypothesis can be further explained that the hippocampal neural stem cells are largely inhibited accomplished with any depressive symptoms in PD conditions. Normally, hippocampal formation of synaptic input from a neuronal cell can extend all the way to the pyramidal cell layer of CA3 region, where established connections of synapses integrate into the trilaminar loop of the hippocampus. PD conditions disrupt their neuronal development by affecting neuronal cell identity with the developing stem/progenitor cell stages, thereby inhibiting majority of differentiating and proliferating neural cells (to be considered that changes in the neural stem/progenitor cells hinder the development of new and mature neurons) integrated into the loop. In addition, the hippocampus develops one of anatomical inputs to the hypothalamus, modifying stress responses under inhibitory negative feedback controls. HPA axis hyperfunction has been presented in around 70% of patients linked to hippocampal changes [70].

In our research hypothesis, relief against the involved psychosocial dysfunction of pre-PD depression is probably based on evidence in adult mammalian models with the decrease (or increase) of hippocampal neuronal regeneration leading to the occurrence of (or recovery from) the depressive-like behaviors. In early PD/pre-PD brains, there are limited regions showing potential pathological changes, as well as showing neurogenesis, e.g., to the glossopharyngeal nerve, the olfactory bulb, or the vagal nerve. Once the symptoms appear, indicated as reparative damaged functions to the hippocampus, cellular protection for hippocampal neurons should be implemented. By study models, it allows direct critical cellular/pathobiological measure underlying anti-depression after promoting hippocampal neuronal regeneration. For the use of chronic continuous intensive stimulation to establish depressive mice, with antidepressant drugs such as fluoxetine or imipramine demonstrated anti-depressive, it is considered appropriate to study the hippocampal involvement. As in the previous study of their hippocampus destroyed by X-ray radiation experimentally, there has been few relieving effects with the same antidepressive intervention [71]. It is then concluded that hippocampal neuronal restoration/regenerative responses (potentially by stem cells) are the basis for antidepressants (neurogenesis that endogenous stem cells provide). Theoretically, the stem cells should also be associated with nonspecific symptomatic relief in the prodromal phase that may ultimately reduce the common severity and improve the quality of life in Parkinson’s patients.

An application on cell therapies may be helpful to meet the expectations for the relief of depressive symptoms in PD [72]. In general, to be tested, MSC (such as bone marrow-derived ones) may inhibit pro-inflammatory microenvironment, for an efficient prohibition of proliferative peripheral blood mononuclear cells or reduction in the infiltration of neutrophils and an activation of microglia around the lesion (intercellular α-synuclein in microglia), and the inflammation-related factors IL-1β, IL-2, and IL-4 around the lesion. The expression of inflammatory factors such as IL-6 and TNF-α is significantly downregulated. When MSC and T cells are cultured within an appropriate ratio, MSC may inhibit T-cell proliferation and modulate pro-inflammatory state, e.g., MSC of the bone marrow derived. In addition, differentiated stem cells can also maintain normal levels of amino acid neurotransmitters in the brains of epileptic mice (inhibition of the progression of epileptogenic process, prophylaxis against the development of chronic epilepsy, and seizure remission) [73]. Cognitive improvement against early PD/pre-PD cognitive deficits has been considered as one of the chronic beneficial effects. In the middle-aged (43–52 weeks) mice model, depressive-like behaviors can be normally observed 4 to 10 months before an onset of PD-like progressive motor dysfunction.

## Discussion

Stem cell transplantation into the depressive brain has been explored/proved in different study models. One example is the hippocampal orthotopic transplantation of MSC populations for the treatment of depression in the mouse model. Their survival time for the stem cells after transplantation may have been relatively short, but the mechanism of its action involves vasculature recruitment (for guiding neuronal migration) to promote endogenous neuronal regeneration. It does improve anxious behavior, depression, learning and memory, and other executive functions [74]. Vascular degeneration can occur and lead to parkinsonism-related cerebral hemorrhage. From our previous work, the preconditioning incubation of stem cells can dramatically improve cellular effects on sensory/motor functional deficits in the mice with intracerebral hemorrhage. There are significantly improved depression-like symptoms following cerebral hemorrhage [75]. Spontaneous range of motion of mice with cerebral hemorrhage is limited, curled up in the corner, and the modification and standing movements are significantly reduced. After treated by nasal transplantation of stem cells, the mice to explore unfamiliar environments can be significantly improved, as well as the number of turns, grooming behaviors, standing up, and other indicators. In our and other group research, stem cells have been utilized for a trial study in mouse models of subarachnoid hemorrhage with improved arachnoid membrane.

Depressive-like symptoms after inferior cavity hemorrhage are relieved with increased expression of tyrosine hydroxylase in the substantia nigra and basal ganglia [76], a new technology allowing the cells particularly suitable for anti-early PD/anti-pre-PD through multiple mechanisms of action. Meanwhile, stem cell transplantation via nasal cavity route is demonstrated safe, effective, and reproducible. The nasal-brain route for any potential methodology of drug delivery has been adopted via the injected stem cells as a way of brain matter targeting. We assume that traditional methods including original displacement around brain tissue lesions implantation, lateral ventricle injection, intrathecal injection, and transplantation in animal models can cause side damage to normal brain tissue/nerve tissue to varying degrees. This noninvasive delivery route of intranasal transplantation may be performed without anesthetic drug interventions and is available for repeatedly injecting and less frequently induced repeated damages. With the advantage of nasal administration, stem cells can bypass the BBB and directly enter the brain matter through the olfactory nerve, perivascular space, trigeminal nerve, etc., reducing the systemic administration-related side effects of stem cells. Other rationale of the nasal/trigeminal pathways for the recently tested mechanisms has included the better feasibility and safety in preclinical studies and clinical trials. Intranasal transplantation of stem cells has been tested as a treatment method against cerebral infarction, cerebral hemorrhage, neonatal cerebral infarction, and other brain vascular diseases. Our group has also published several preclinical studies with stem cells transplanted through the nasal cavity. The cells enter the brain, being distributed around the ischemic or hemorrhagic lesions with improved neurological symptoms after stroke [77].

**Fig. 2:**
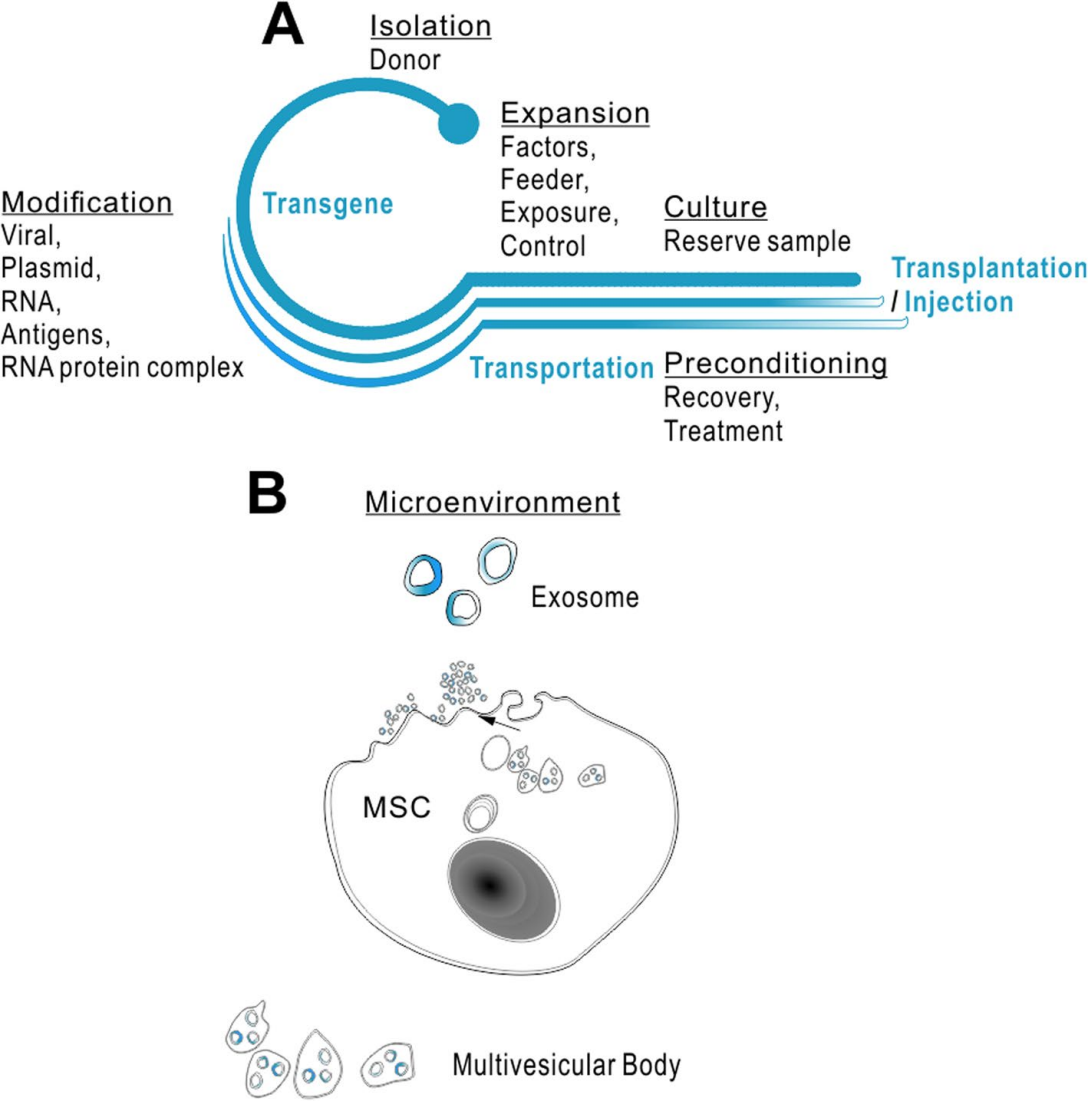
Exosomes mediated microenvironment-based strategies by stem cells. Types of tissue stem cells have been collected from adipose tissue, amniotic fluid, bone marrow, olfactory mucosa, peripheral blood, placenta, umbilical cord blood, and urine. Included stem cells are from multi-differentiation capacity, from well proliferation and differentiation ability, from the potentials of binding biomaterials, from the ease of isolation and culture, or from the non-immunogenicity of autologous transplantation. It has been attempted to summarize an outline for an action of transplantation therapy following the cell treatment against PD [80]. In tissue engineering and regenerative medicine, hUC-MSC that are isolated from human umbilical vein endothelium and subendothelial layer have been broadly approved [81]. Endogenously, MSC exist in connective tissues and interstitium of organs throughout the body. Tissue-derived MSC are one of the important types of adult stem cells, by in vitro conditions, which are shown with self-renewal, proliferation, and multi-differentiation potentials. In the in vitro condition, MSC demonstrate adherent and migrating growth capacity, shown with relatively uniform spindle cells or polygonal cells (hold G0–G1 phase of cell cycle for 80% after 30 passages) presenting normal diploid/maintaining the morphology and proliferation characteristics. MSC highly express CD73, CD90, and CD105 (human gene name: *NT5E*, *THY1*, *ENG*) with adhesion molecule markers such as CD13, CD29, CD44, and CD54 and express low levels of MHC-I molecular markers such as HLA-ABC but show negative for CD34 or CD45, and divergently, for CD31 and other hematopoietic stem cell markers and HLA-DR, HLA-DA, HLA-DP, HLA-DQ, and other MHC-II molecular markers [82]. MSC can express with OCT-4 (human gene name: *POU5F1*), an embryonic stem cell-characterized marker gene. Specifically, OCT-4/SOX2 in UC-MSC is closely related to maintaining the undifferentiated state of stem cells. Regulation via transcriptional controlling directed multi-lineage differentiation may have included roles in CEBPA gene family members, COL2A1/10A1, DLX5, OPN, OSX (lncRNA MALAT1 pairing), PPARG, RUNX2, SOX9, and Wnt gene family members, of epigenetic mechanisms involving ASH1L, EHMT1/ESET, HDAC1/6/8, KDM4B/6A/6B, or SIRT1/2. Some sorts of exosomes (such as enriched with lncRNA RUNX2-AS1 pairing derived from UC-MSC cell types towards osteogenic differentiation) are particularly useful in the clinical setting as hoped for microenvironment-based targeting of cardiomyocytes, chondrocytes, endothelial cells, germ cells, hepatocytes, neural cells (including glial cells), osteoblasts, skeletal muscle cells, or tumor cells. In addition, application of MSC may possess strong phenotypic plasticity and particularly low immunogenicity

**Fig. 3:**
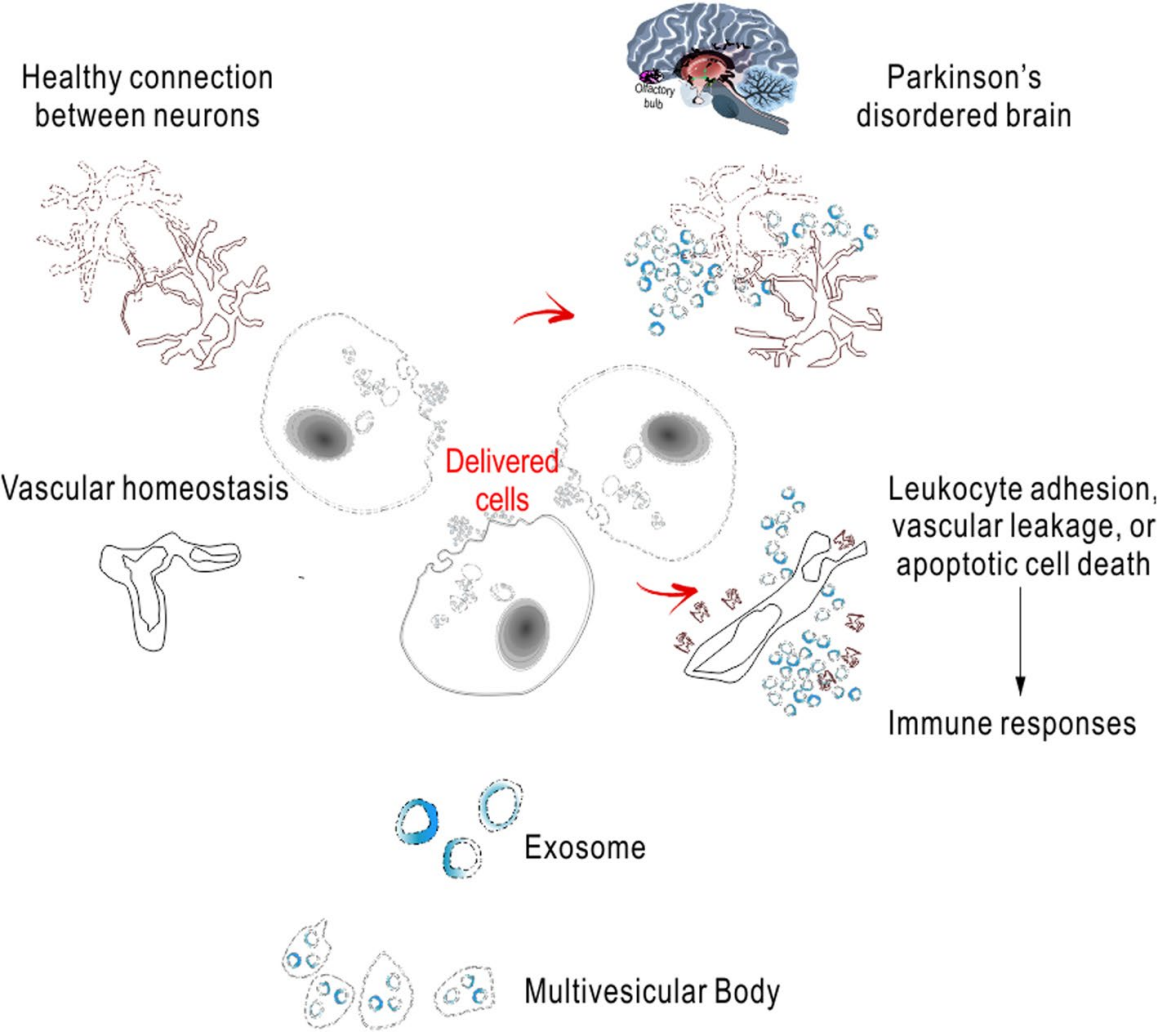
Biotherapeutic disease-modifying treatment. The specific PD-NMS treatment outcome evaluation is included in the 2011 and 2019 guidelines of the International Parkinson’s Disease and Movement Disorders Society (www.movementdisorders.org), providing their optimized, recommended, and standardized treatment strategic issues. It has been performed based on the clinical significance, the effectiveness, and the safety, for any random trials on large cohort of PD-NMS intervention candidates only. By number, PD becomes the second most common progressive neurodegenerative disease, demonstrated by a variety of pathological features with the progressive cellular loss for substantia nigra dopaminergic neurons and by an appearance of neurotoxic Lewy bodies. To our knowledge, whether precise neuronal cell replacement strategies may halt the disease progress/delay the PD symptoms remains unproved. In addition to the typical manifestations with PD motor symptoms, there is, more devastating if untreated, a wider range of NMS to be managed [83]. Since that occurrence usually lasts over years, all together, PD and related symptoms can bring huge costs and problems. There are major impacts on patient families, hospitalization/nursing cost, and challenge to the clinical management [84]. It is critical for identifying an early PD/pre-PD stage with PD-NMS considering diagnosis/treatment. PD-NMS with/without symptomatic comorbidities usually involve larger individual differences and wider spectrum from lack of specificity. Those symptoms are potentially driven by underlying disease processes rather than coping with PD. Fundamental to PD-NMS, many of which begin prior to motor deficits, the pathological feature distinctly involves non-dopaminergic processes. Current PD treatments against both its motor symptoms and PD-NMS as well as considerations for an effective recognition of PD-NMS management are referenced in the “Chinese Parkinson's Disease Treatment Guidelines,” [85] recently updated with the fourth edition

Recently, our and others lab’s published results support hypothesis with intranasally delivered stem cells (e.g., MSC, iPSC-derived cells) to improve the depressive-like symptoms as a consequence of inhibiting brain inflammation and/or promoting endogenous neuronal regeneration and repair. Specifically, the 8-week-long chronic unpredictable mild stress (CUMS) of mice is established, which induces stable depressive-like phenotype under hyperactive HPA axis but no later than 36 days without any further CUMS treatment [78]. In order to verify the above hypothesis, our standard protocols include labeling procedures in vitro, prior to nasally transplanting. In experimental design, evaluation groups are given fluoxetine for the control treatment of positive responses and then following transplantation perform dynamically tracing (under NMR if use the SiPO-labeled cells for transplantation). The data provides evidence for the cell retention and endogenous glial/neuronal regeneration. It will be further expected for the involvement of anti-inflammatory responses, neuronal regenerative, or some other molecular signaling (BDNF-TrkB-PI3K/Akt, β-catenin, and Notch) pathways providing mechanisms for the additional beneficial effects to the depression (Fig. 2) [79]. Update results support that the stem cell transplantation improves host brain’s conditions and is demonstrated safe and promising for pain management and the treatment of anxiety, depression, and insomnia or other PD premotor symptoms (Fig. 3).

## Conclusions

The study reported is considered important and relevant for update on Parkinson’s interventions as well as for biotherapy-related education. Among them, stem cell therapy may be one particularly effective outcome-improving strategies in PD-NMS treatment at their early PD/pre-PD stages via cell replacement or vascular mechanisms. Underlying PD-NMS such as depression and neuroimmune modulation can be accompanied alongside the host microenvironmental improvement by mesenchymal stromal cell (including exosome) biotherapy within the brain of PD conditions of disrupted neuronal connections and vascular homeostasis. Consequently, neuro-replacing and vascular component repairs are expected as linked between objectives and the performance of its beneficial effects.

## Data Availability

Data sharing is not applicable to this article as no dataset has been generated or analyzed during the current study. For any shared data potentially from publicly accessible resources, please use the lead contact on information request for the CtrLyin Group by fax (no. + 1 912–590-0223) or email at WeiZZ@ctrlyin.org (Z. W.).

## Abbreviations

BDI: The Beck Depression Inventory
BDNF: Brain-derived neurotrophic factor
CBT: Cognitive-behavioral therapy
CUMS: Chronic unpredictable mild stress
HAM-A: The Hamilton Anxiety Rating Scale
HPA: Hypothalamic pituitary adrenal
HSPC: Hematopoietic stem and progenitor cell
MAO-BI: Monoamine oxidase-B inhibitor
MDS: International Parkinson and Movement Disorder Society
MHC: The major histocompatibility complex
MSC: Multipotent/mesenchymal stromal cells
NMR: Nuclear magnetic resonance
NMS: Non-motor/premotor symptoms
NMSS: The non-motor symptom scale
RBD: REM sleep behavior disorder
REM: Rapid-eye movement
SCF: Stem cell factor
UC: Umbilical cord or umbilical cord derived
VEGF: Vascular endothelial growth factor
